# Supplementary material for: Structure-Based Designing, Solvent Less Synthesis of 1,2,3,4-Tetrahydropyrimidine-5-carboxylate Derivatives: A Combined In Vitro and In Silico Screening Approach
Source: Molecules. 2021 Jul 22;26(15):4424. doi: 10.3390/molecules26154424 (PMC8348070; doi:10.3390/molecules26154424)
Supplement: Supplementary file 1 [file molecules-26-04424-s001.zip › molecules-1264321-supplementary.pdf]

Supplementary Materials

List of Figures for Docking Interaction

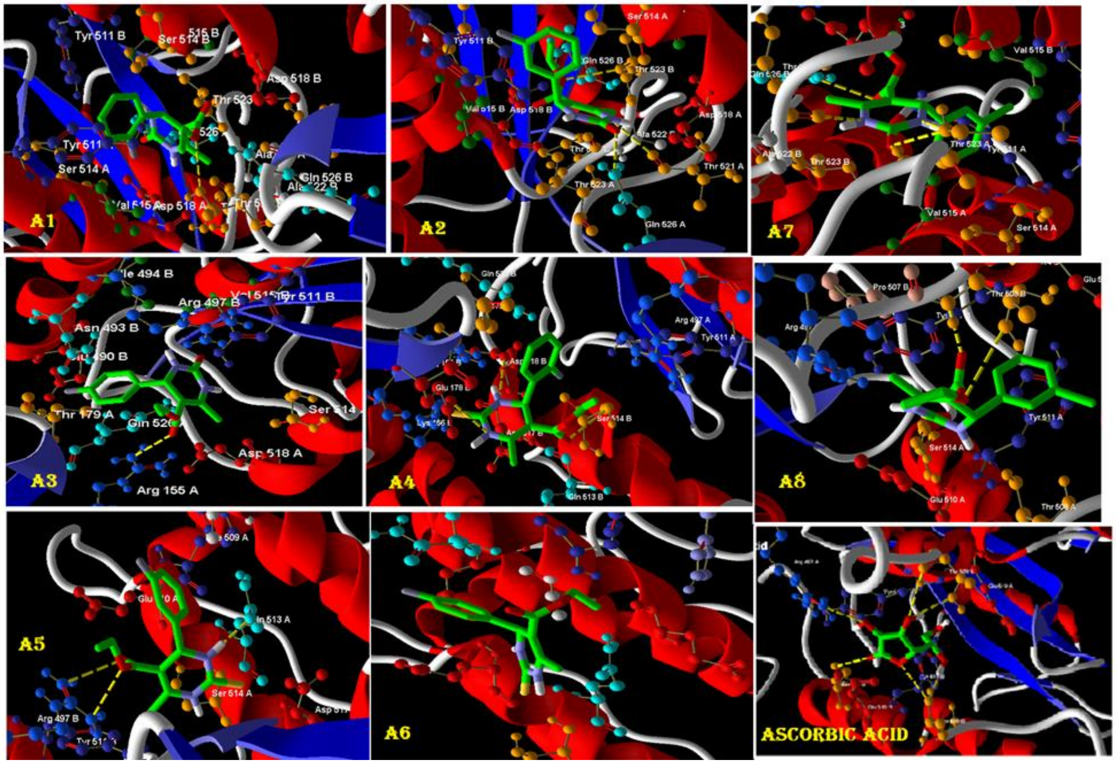

Figure S1. (A): Leading pose of compounds **5a-h** into binding site of 2HCK for antioxidant activity along with standard drug Ascorbic acid: (**A1**) coupling of compound **5a** with residues of 2HCK; (**A2**) coupling of compound **5b** with residues of 2HCK; (**A3**) coupling of compound **5c** with residues of 2HCK; (**A4**) coupling of compound **5d** with residues of 2HCK; (**A5**) coupling of compound **5e** with residues of 2HCK; (**A6**) coupling of compound **5f** with residues of 2HCK; (**A7**) coupling of compound **5g** with residues of 2HCK; (**A8**) coupling of compound **5h** with residues of 2HCK; Coupling of Ascorbic acid with residues of 2HCK.

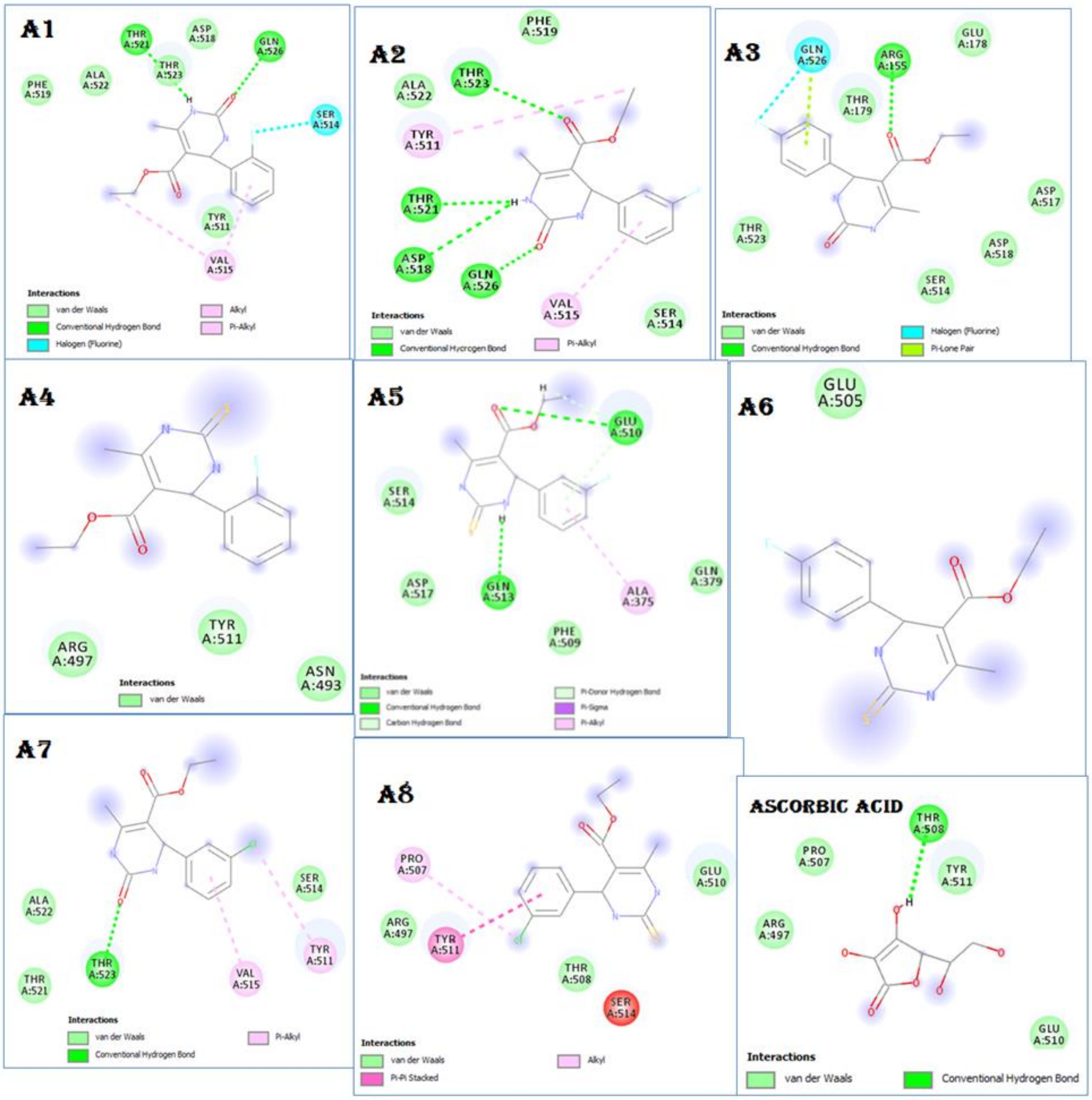

Figure S1. (B): 2D visualization of compound **5a-h** by Discovery Studio Visualizer; (**A1**) 2D model of compound **5a**; (**B2**) 2D model of compound **5b**; (**A3**) 2D model of compound **5c**; (**A4**) 2D model of compound **5d;** (**A5**) 2D model of compound **5e;** (**A6**) 2D model of compound **5f;** (**A7**) 2D model of compound **5g;** (**A8**) 2D model of compound **5h** and Ascorbic acid a standard drug.

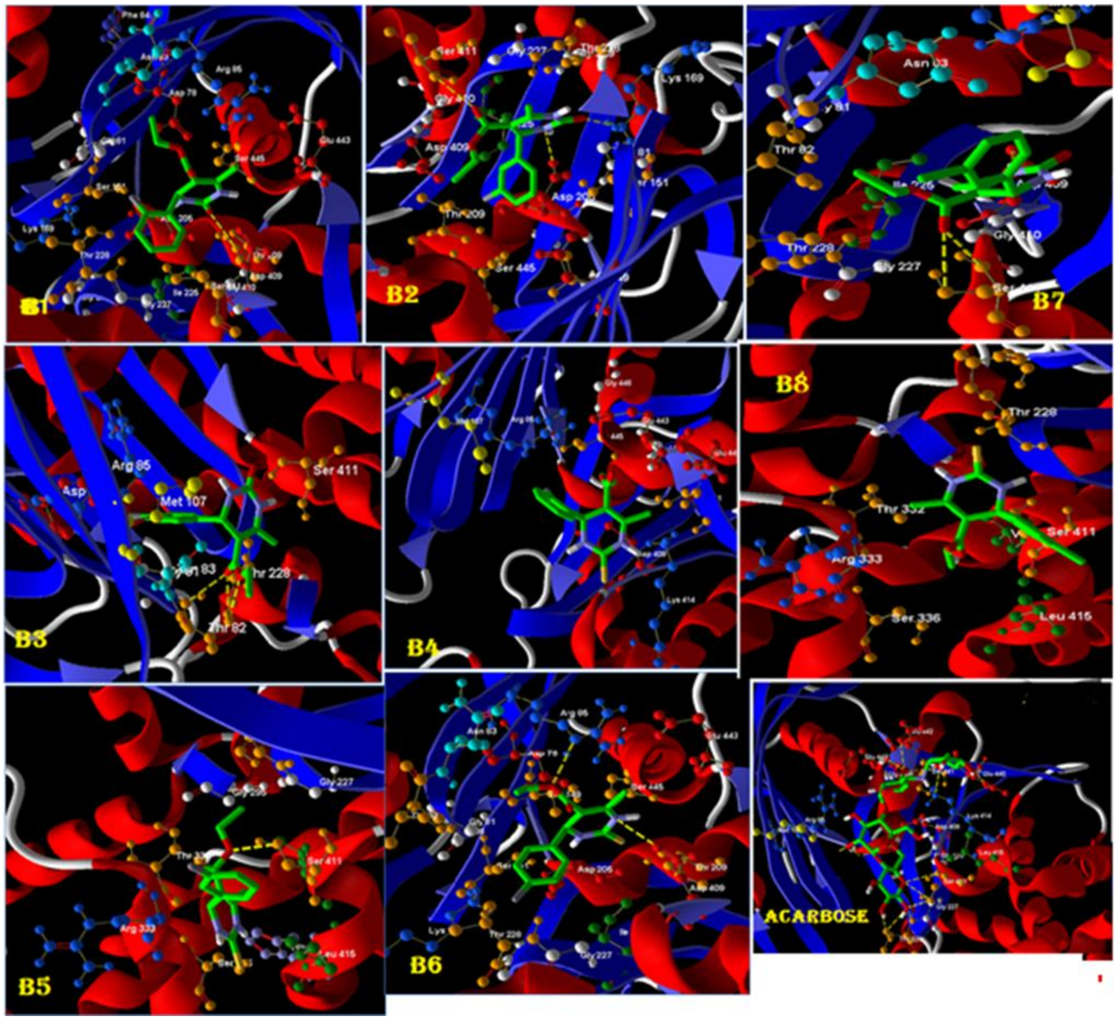

Figure S2. (A): Leading pose of compounds **5a-h** into binding site and standard drug Acarbose into binding site of 1V4S for anti-diabetic activity; **(B1)** binding mode of compound **5a** into 1V4S (**B2**) binding mode of compound **5b** into 1V4S; **(B3)** binding mode of compound **5c** into 1V4S; **(B4)** binding mode of compound **5d** into 1V4S; **(B5)** binding mode of compound **5e** into 1V4S; **(B6)** binding mode of compound **5f** into 1V4S; **(B7)** binding mode of compound **5g** into 1V4S; **(B8)** binding mode of compound **5h** into 1V4S; Binding mode of **acarbose** into 1V4S.

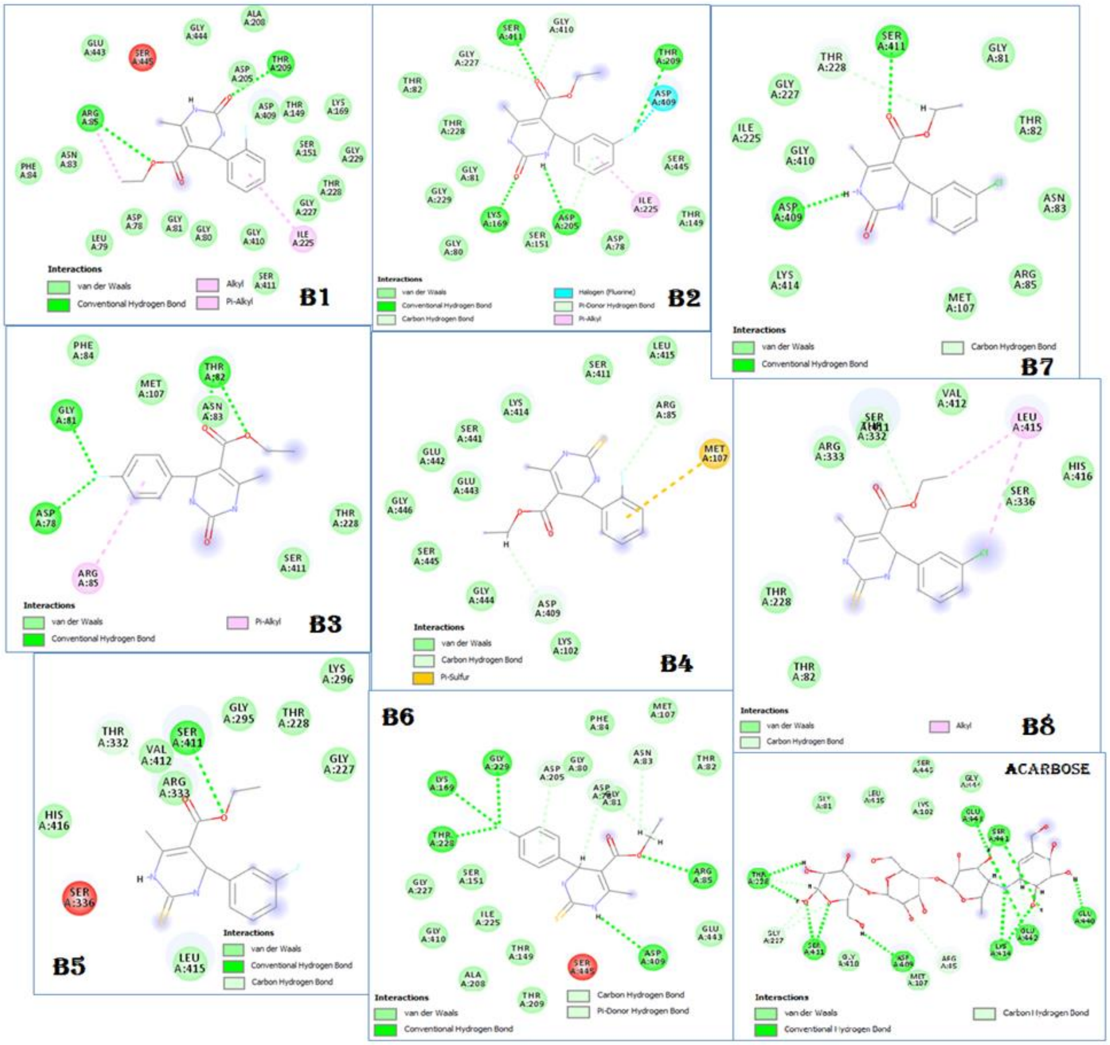

Figure S2. (B): 2D visualization of compound **5a-h** by Discovery Studio Visualizer; (**B1**) 2D model of compound **5a**; (**B2**) 2D model of compound **5b**; (**B3**) 2D model of compound **5c**; (**B4**) 2D model of compound **5d;** (**B5**) 2D model of compound **5e;** (**B6**) 2D model of compound **5f;** (**B7**) 2D model of compound **5g;** (**B8**) 2D model of compound **5h; ACARBOSE** a standard drug.

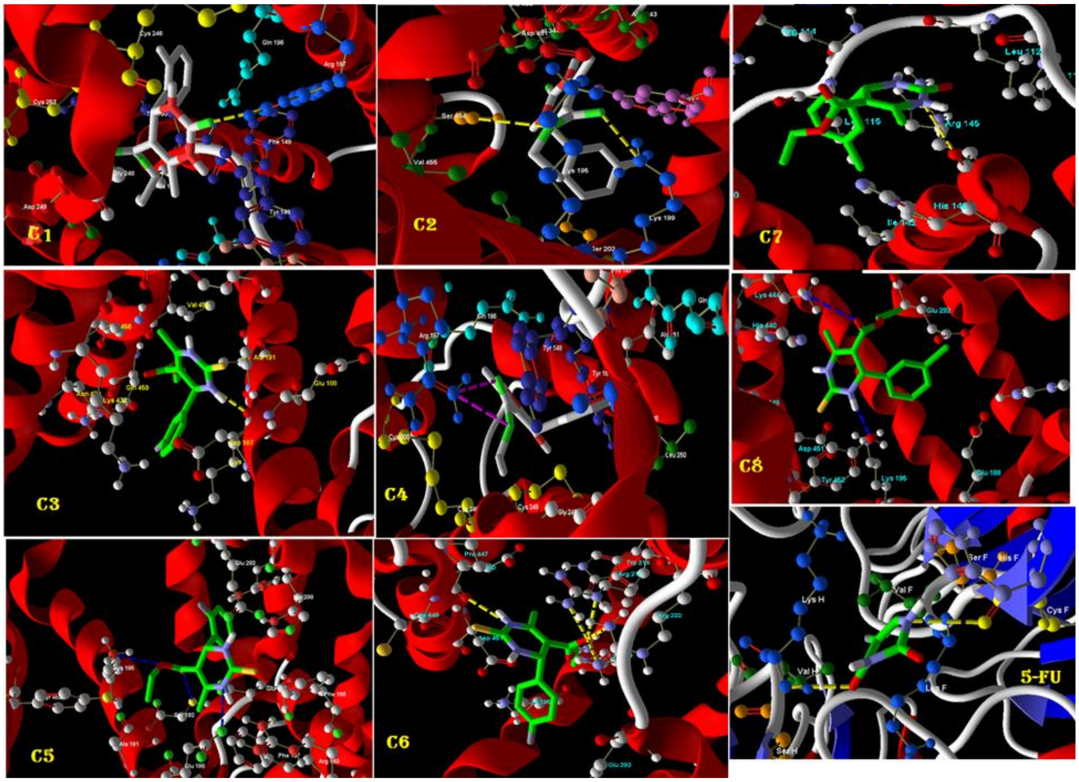

Figure S3. (A): Leading pose of compounds **5a-h** into binding site of 3B9L for anti-cancer activity**:** (**C1**) coupling of compound **5a** with residues of 3B9L; (**C2**) coupling of compound **5b** with residues of 3B9L; (**C3**) coupling of compound **5c** with residues of 3B9L; (**C4**) coupling of compound **5d** with residues of 3B9L; (**C5**) coupling of compound **5e** with residues of 3B9L; (**C6**) coupling of compound **5f** with residues of 3B9L; (**C7**) coupling of compound **5g** with residues of 3B9L; (**C8**) coupling of compound **5h** with residues of 3B9L; Coupling of 5-FLOURO-URACIL with residues of 3B9L.

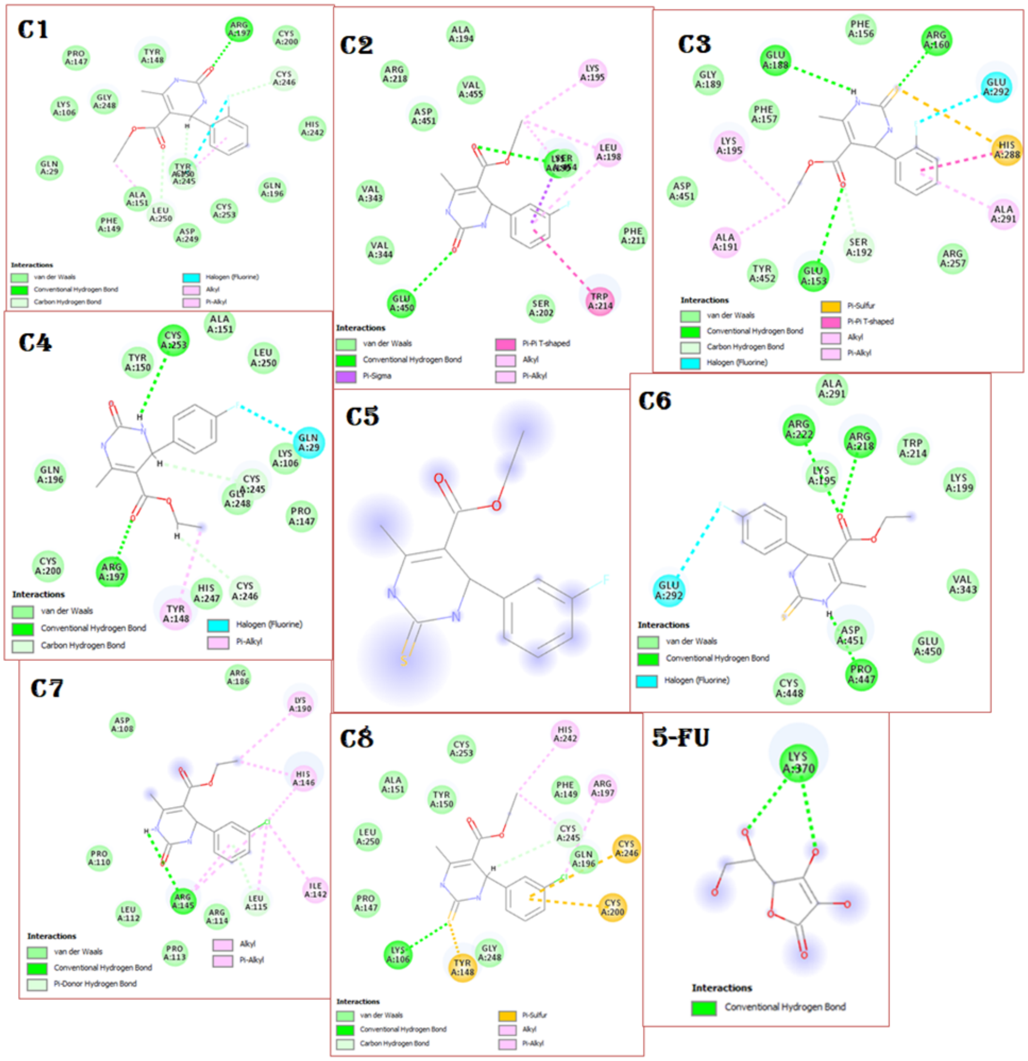

Figure S3. (B): 2D visualization of compound **5a-h** by Discovery Studio Visualizer; (**C1**) 2D model of compound **5a**; (**C2**) 2D model of compound **5b**; (**C3**) 2D model of compound **5c**; (**C4**) 2D model of compound **5d;** (**C5**) 2D model of compound **5e;** (**C6**) 2D model of compound **5f;** (**C7**) 2D model of compound **5g;** (**C8**) 2D model of compound **5h**; **5-Flourouracil** a standard drug.

List of tables for Docking interaction

Table S1. Molecular docking results of synthesized compounds interacting amino acids residues in the active site of carboxypeptidase D isoform (PDB ID: 2HCK) as anti-oxidant agents.

Compounds	Mol dock score (kcal/mol)	No of hydrogen bonds	Amino acid involved in bonding	Atoms bonded to amino acid	Hydrogen bond length Å
Ascorbic acid	-81.405	5	Ser 513 (C=O)	Carbonyl oxygen	3.26
			Thr 508 (O)	Oxygen of γ-lactone	3.18
			Arg 497 (OH)	9-OH	2.93
			Thr 508 (OH)	10-OH	3.30
			Thr 508 (OH)	11-OH	3.18
**5a**	-102.409	2	Thr 523 (O)	Ester linked O	3.05
			Gln 526 (C=O)	O of oxo group	2.61
**5b**	-109.978	3	Thr 523 (O)	Ester linked O	3.11
			Asp 518 (NH)	1-NH	3.50
			Gln 526 (C=O)	O of oxo group	2.5
**5c**	-132.323	1	Arg 155 (C=O)	O of ester linked carbonyl	2.967
**5d**	-92.51	3	Ser 514 (C=O)	O of ester linked carbonyl	3.06
			Glu 178 (NH)	1-NH	3.44
			Asp 518 (NH)	3-NH	3.40
**5e**	-123.923	3	Arg 497 (O)	Ester linked O	3.27
			Arg 497 (C=O)	O of ester linked carbonyl	3.21
			Gln 513 (NH)	3-NH	2.96
**5f**	-94.11	0	0	0	0
**5g**	-108.57	3	Gln 526 (C=O)	O of ester linked carbonyl	2.97
			Thr 523 (NH)	3-NH	2.88
			Thr 523 (C=O)	O of oxo group	3.01
**5h**	-115.2	2	Thr 508 (O)	Ester linked O	3.42
			Thr 508 (C=O)	O of ester linked carbonyl	2.57

Table S2. Molecular docking results of synthesized compounds interacting amino acids residues in the active site of human glucokinase (PDB ID: 1V4S) as antidiabetic.

Compounds	Mol dock score (kcal/mol)	No of hydrogen bonds	Amino acid involved in bonding	Atoms bonded to amino acid	Hydrogen bond length Å
Acarbose	-95.8957	11	Thr 228 (OH)<br>Thr 228 (OH)<br>Ser 411 (OH)<br>Ser 411 (O)<br>Asp409 (OH)<br>Glu 22 (OH)<br>Glu443 (OH)<br>Ser 441 (OH)<br>Glu440 (OH)<br>Ser 441 (OH)<br>Lys 414 (OH)	21-OH<br>23-OH<br>23-OH<br>24-O<br>27-OH<br>31-OH<br>31-OH<br>31-OH<br>42-OH<br>44-OH<br>44-OH	2.98<br>2.97<br>2.83<br>3.20<br>2.96<br>3.15<br>2.60<br>2.63<br>2.57<br>2.65<br>2.73
**5a**	-100.867	1	Thr 209 (C=O)	O of oxo group	3.18
**5b**	-107.88	3	Lys 169 (C=O)<br>Ser 411 (C=O)<br>Asp 205(NH)	O of oxo group<br>O of ester linked carbonyl<br>1-NH	2.59<br>2.85<br>3.27
**5c**	-125.71	3	Thr 82 (C=O)<br>Thr 82 (C=O)<br>Thr 82 (O)	O of ester linked carbonyl<br>O of ester linked carbonyl<br>Ester linked O	2.79<br>2.90<br>2.78
**5d**	-104.3	0	0	0	0
**5e**	-100.175	1	Ser 411 (O)	Ester linked O	2.61
**5f**	-98.9958	1	Arg 85 (O)	Ester linked O	3.61
**5g**	-129.805	2	Asp 409 (NH)<br>Ser 41 (C=O)	3-NH<br>O of ester linked carbonyl	2.76<br>3.40
**5h**	-118.135	0	0	0	0

Table S3. Molecular docking results of synthesized compounds interacting amino acids residues in the active site of human serum albumin (PDB ID: 3B9L) as anti-cancer.

Compounds	Mol dock score (kcal/mol)	No of hydrogen bonds	Amino acid involved in bonding	Atoms bonded to amino acid	Hydrogen bond length Å
**5-florouracil**	-65.029	2	Gln29<br>ALA151	O of carbonyl<br>N of amide	3.36<br>2.60
**5a**	-121.701	1	Arg 197 (C=O)	O of oxo group	2.48
**5b**	-118.405	2	Ser 454 (O)<br>Lys 199 (C=O)	Ester linked O<br>O of ester linked carbonyl	3.31<br>2.87
**5c**	-126.332	2	Arg 197 (O)<br>Arg 197 (C=O)	Ester linked O<br>O of ester linked carbonyl	3.21<br>2.66
**5d**	-105.888	3	Ser192 (C=O)<br>Glu188 (NH)<br>Lys195(O)	O of ester linked carbonyl<br>3-NH<br>Ester linked O	2.906<br>3.547<br>3.129
**5e**	-104.162	1	Asp187 (NH)	1-NH	2.601
**5f**	-98.448	5	Arg222 (C=O)<br>Arg222 (C=O)<br>Arg218 ((C=O)<br>Arg218(C=O)<br>Pro447 (NH)	O of ester linked carbonyl<br>O of ester linked carbonyl<br>3-NH	3.436<br>3.322<br>2.773<br>3.442<br>3.284
**5g**	-100.536	1	Arg145 (NH)	3-NH	2.960
**5h**	-108.134	0	0	0	0

1. NMR spectra of 4-(2-fluorophenyl)-6-methyl-2oxo-1,2,3,4-tetrahydropyrimidine-5-carboxylate (5a, $C_{14}H_{15}FN_2O_3$):

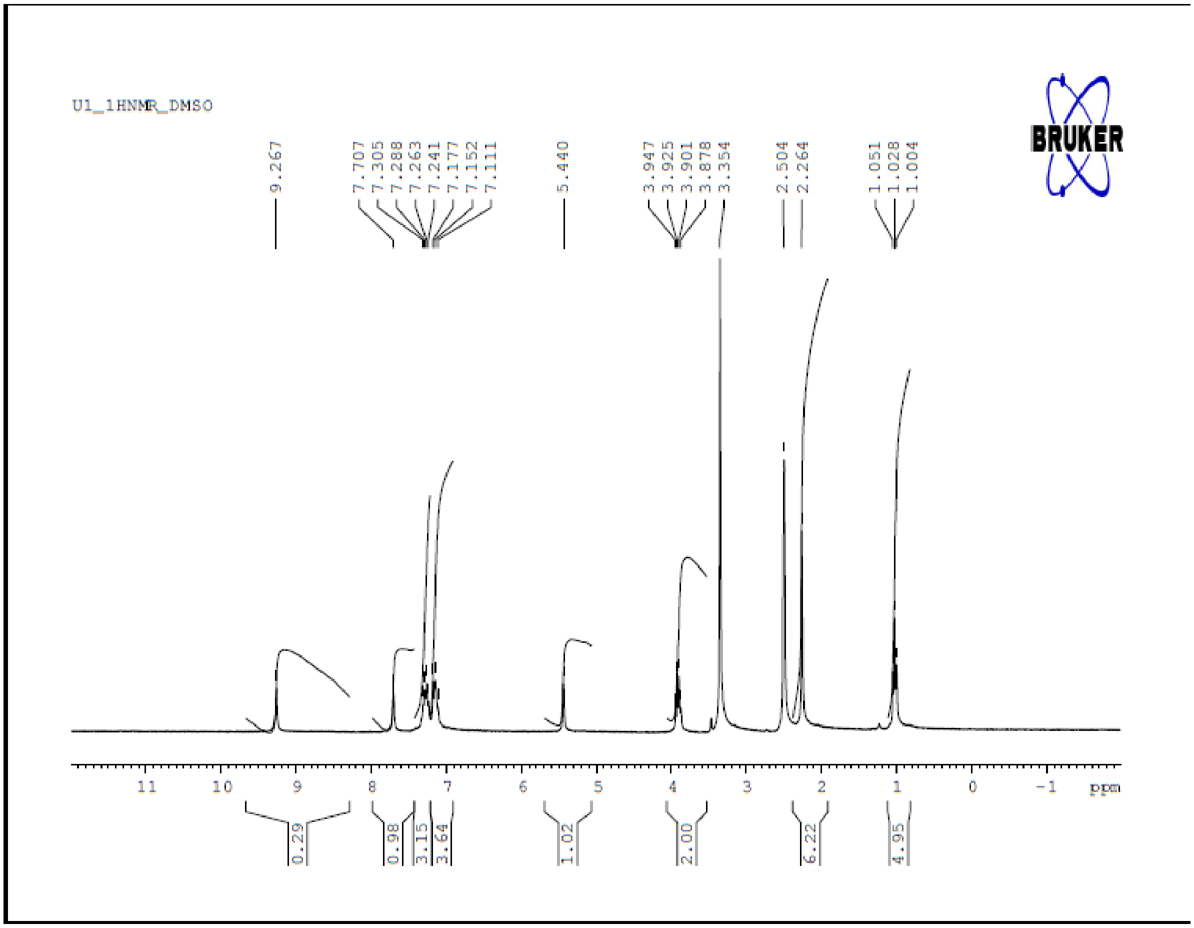

Figure. S4. ^{1}H-NMR spectra of 5a.

2. NMR spectra of ethyl 4-(3-fluorophenyl)-6-methyl-2oxo-1,2,3,4-tetrahydropyrimidine-5-carboxylate (5b, $C_{14}H_{15}FN_2O_3$):

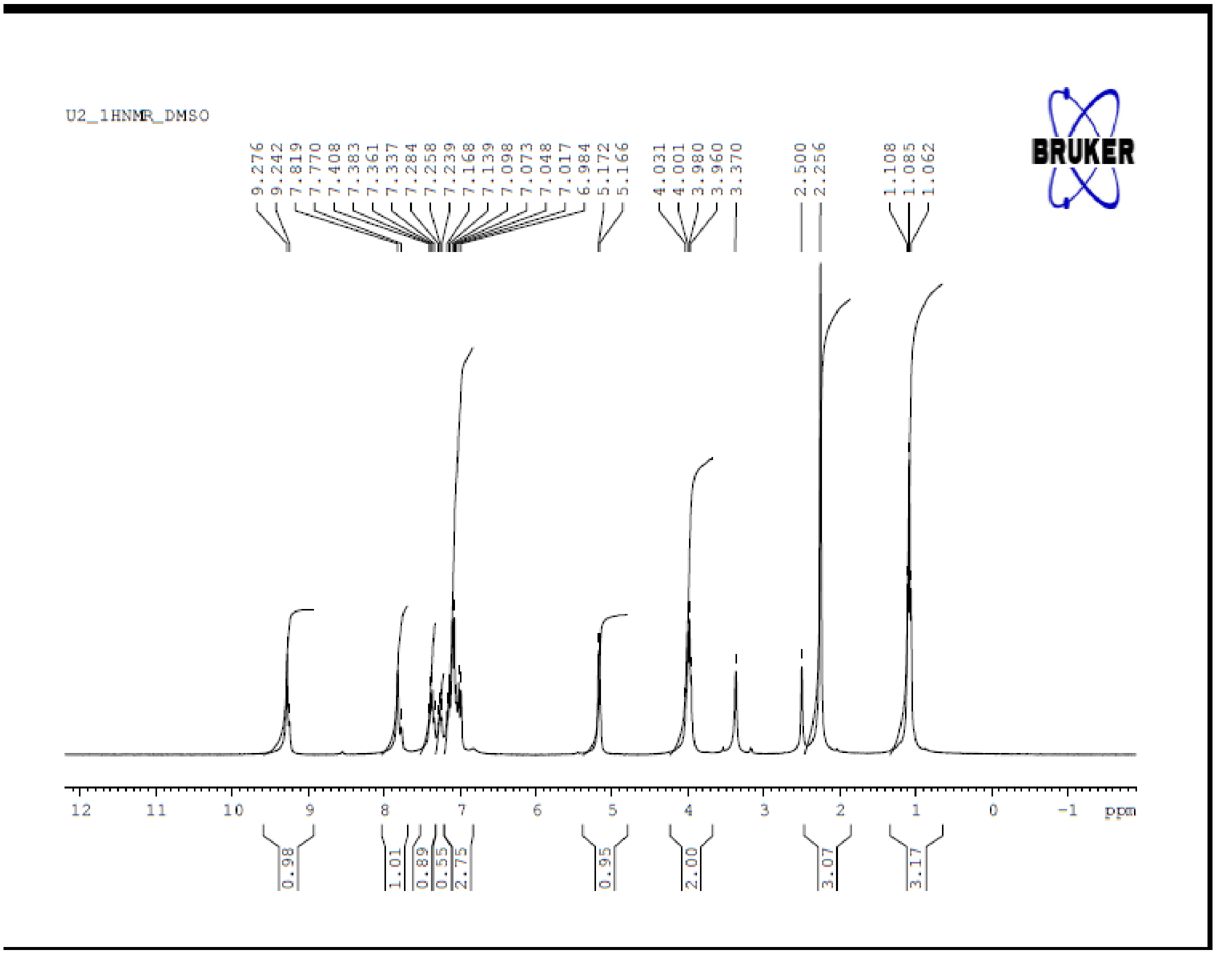

Figure. S5. ^{1}H-NMR spectra of 5b.

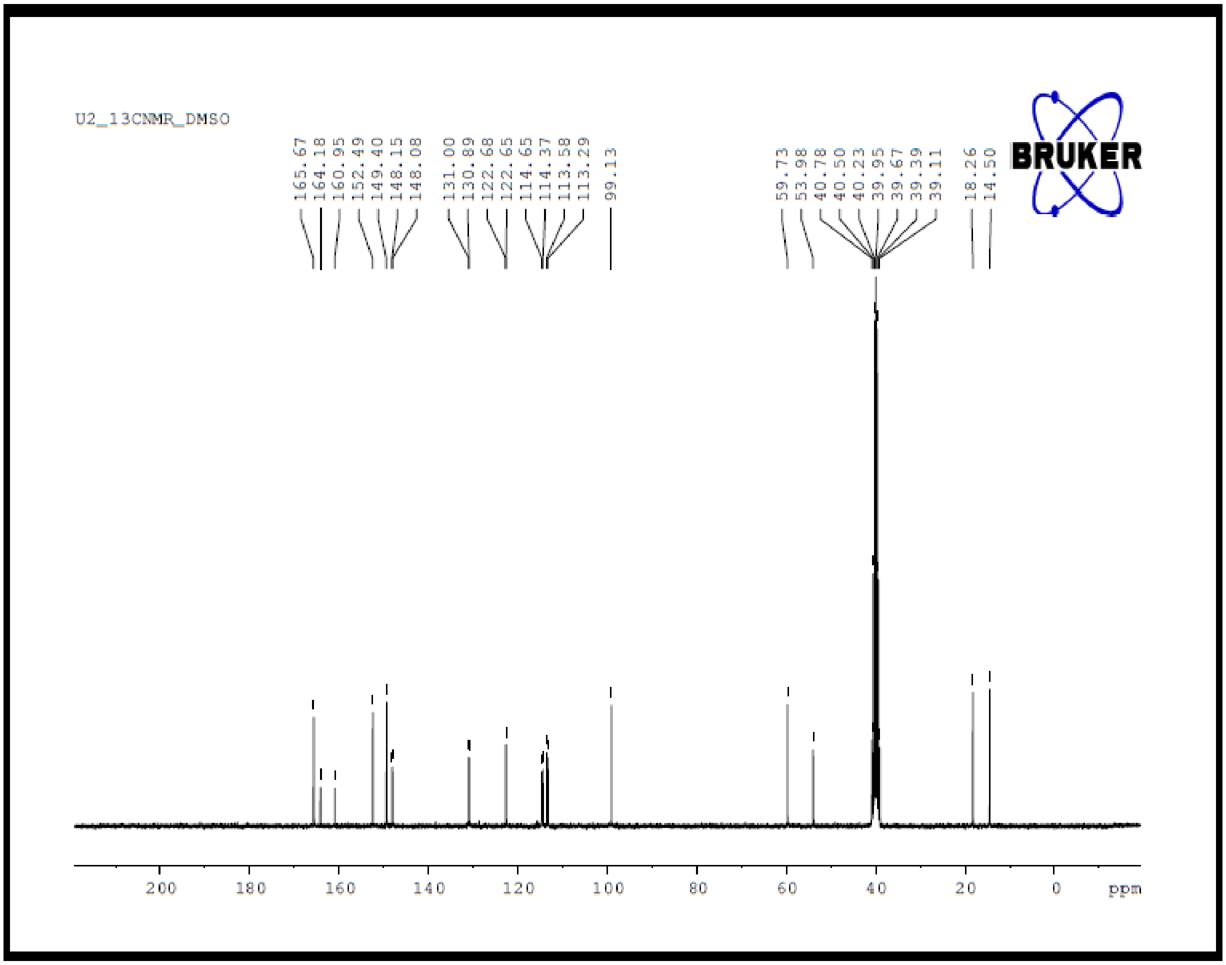

Figure. S6. ^{13}C-NMR spectra of 5b.

3. NMR spectra of ethyl 4-(4-fluorophenyl)-6-methyl-2oxo-1,2,3,4-tetrahydropyrimidine-5-carboxylate (5c, $C_{14}H_{15}FN_2O_3$):

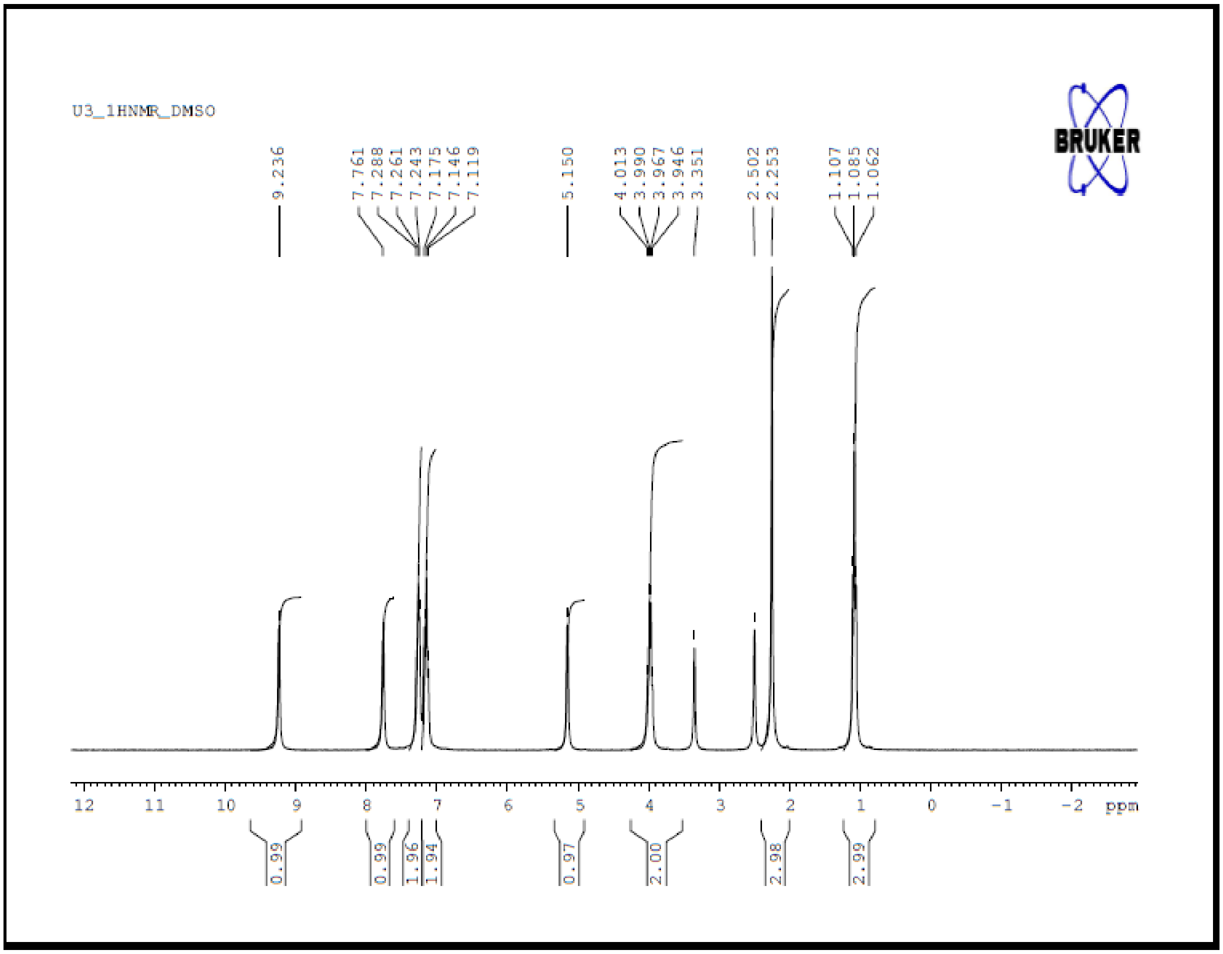

Figure. S7. ^{1}H-NMR spectra of 5c.

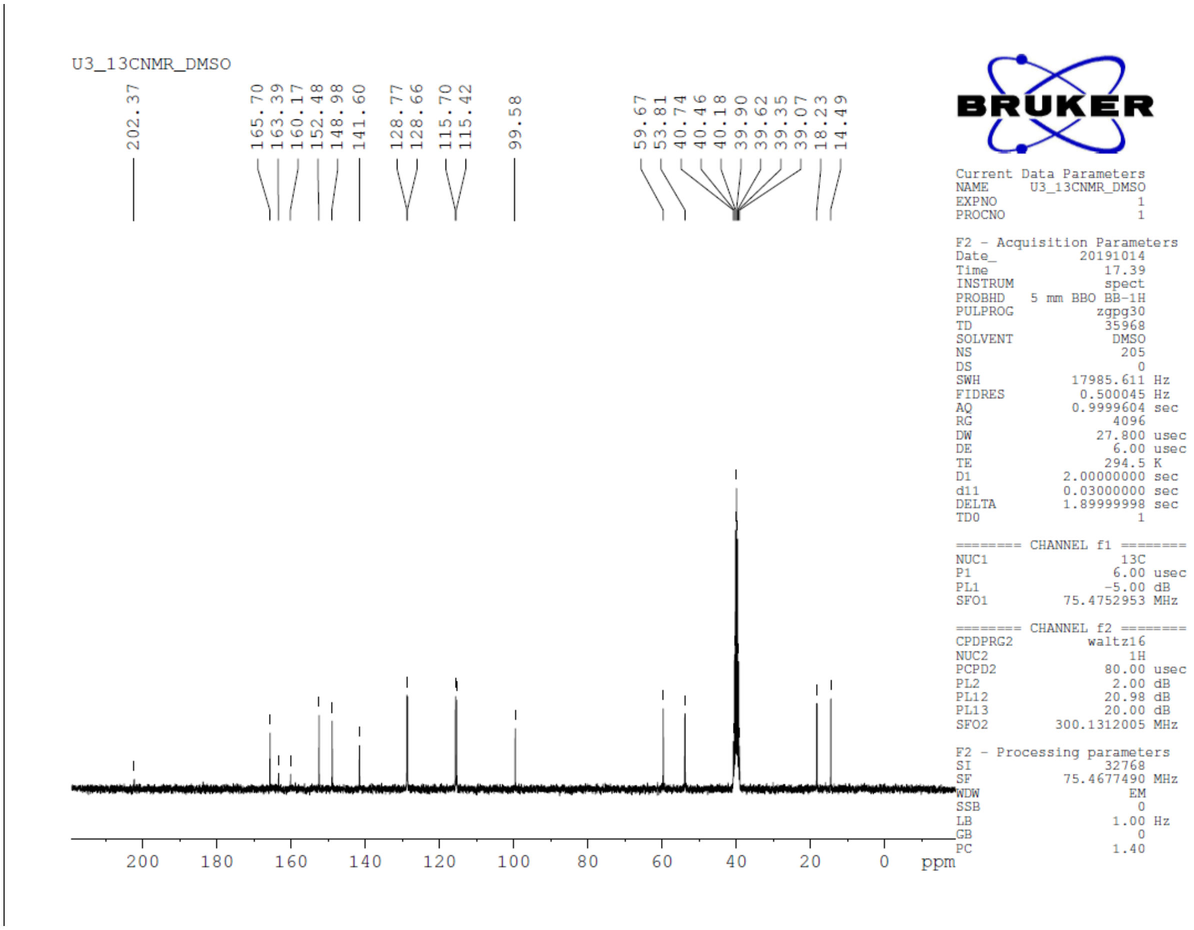

Figure. S8. ^{13}C-NMR spectra of 5c.

4. NMR spectra of ethyl 4-(2-fluorophenyl)-6-methyl-2-thioxo-1,2,3,4-tetrahydropyrimidine-5-carboxylate (5d, $C_{14}H_{15}FN_2O_2S$):

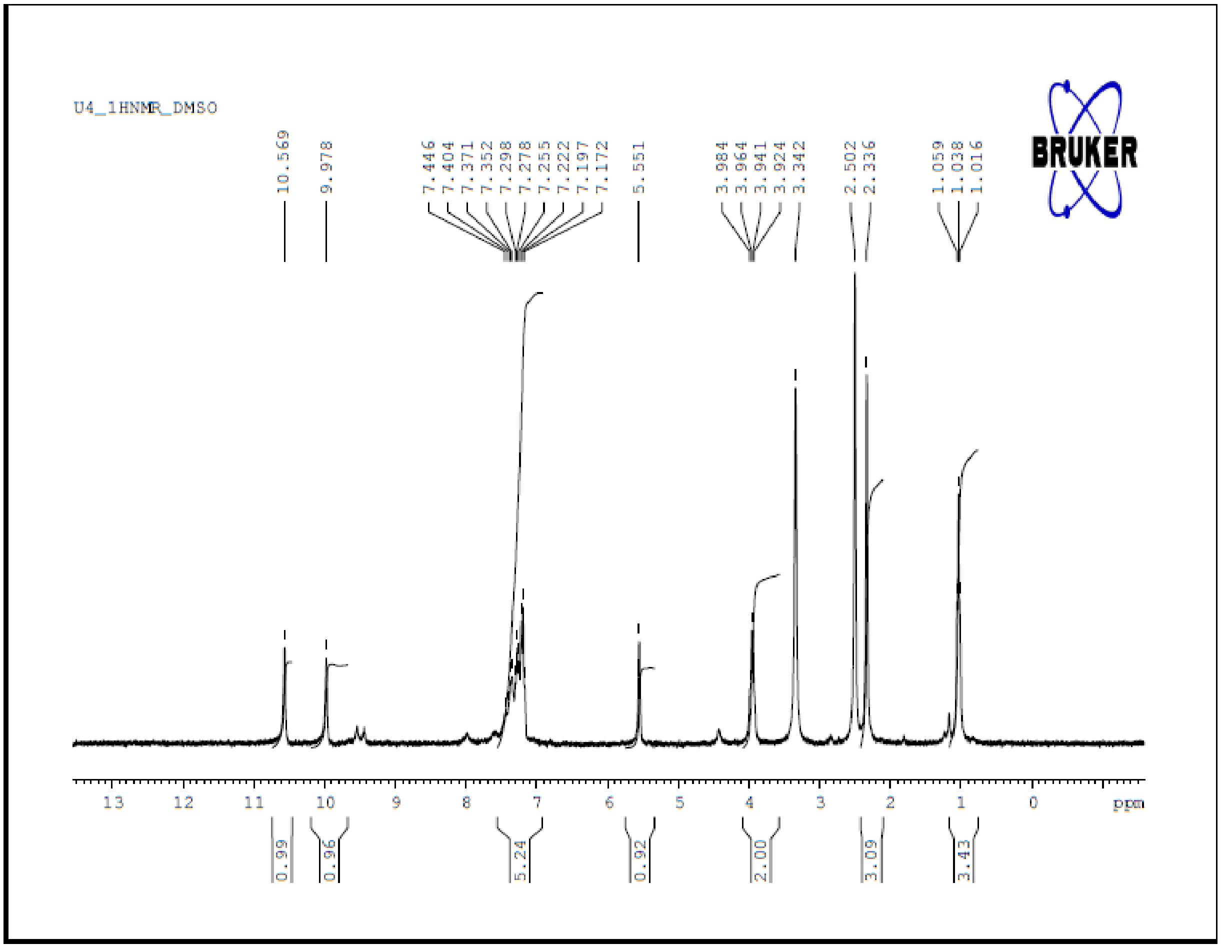

Figure. S9. ^{1}H-NMR spectra of 5d.

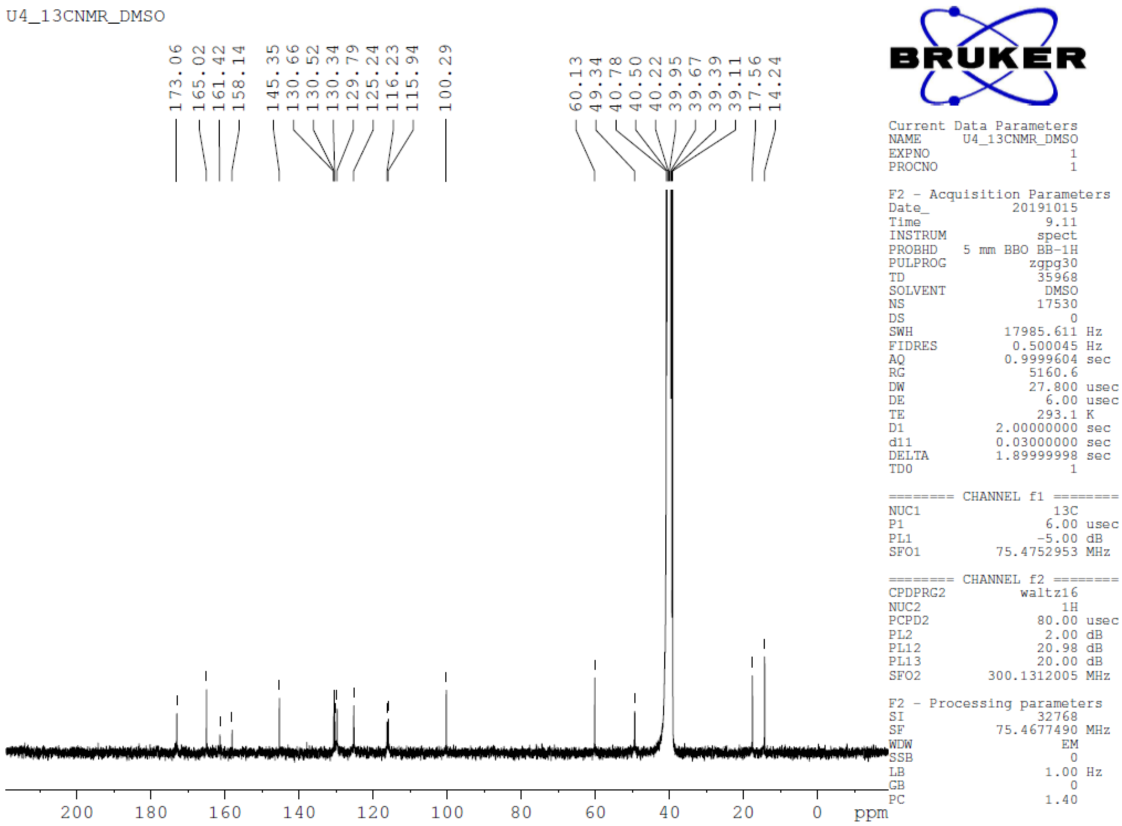

Figure. S10. ^{13}C-NMR spectra of 5d.

5. NMR spectra of ethyl 4-(3-fluorophenyl)-6-methyl-2-thioxo-1,2,3,4-tetrahydropyrimidine-5-carboxylate (5e, $C_{14}H_{15}FN_2O_2S$):

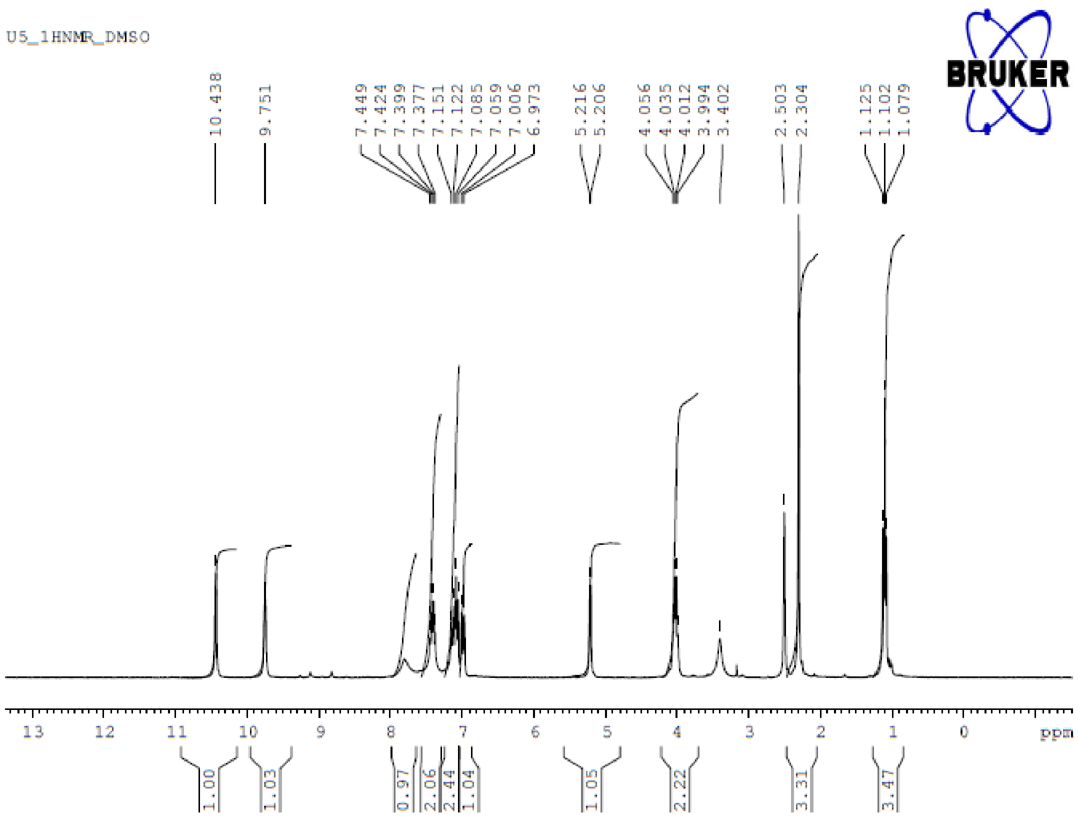

Figure. S11. ^{1}H-NMR spectra of 5e.

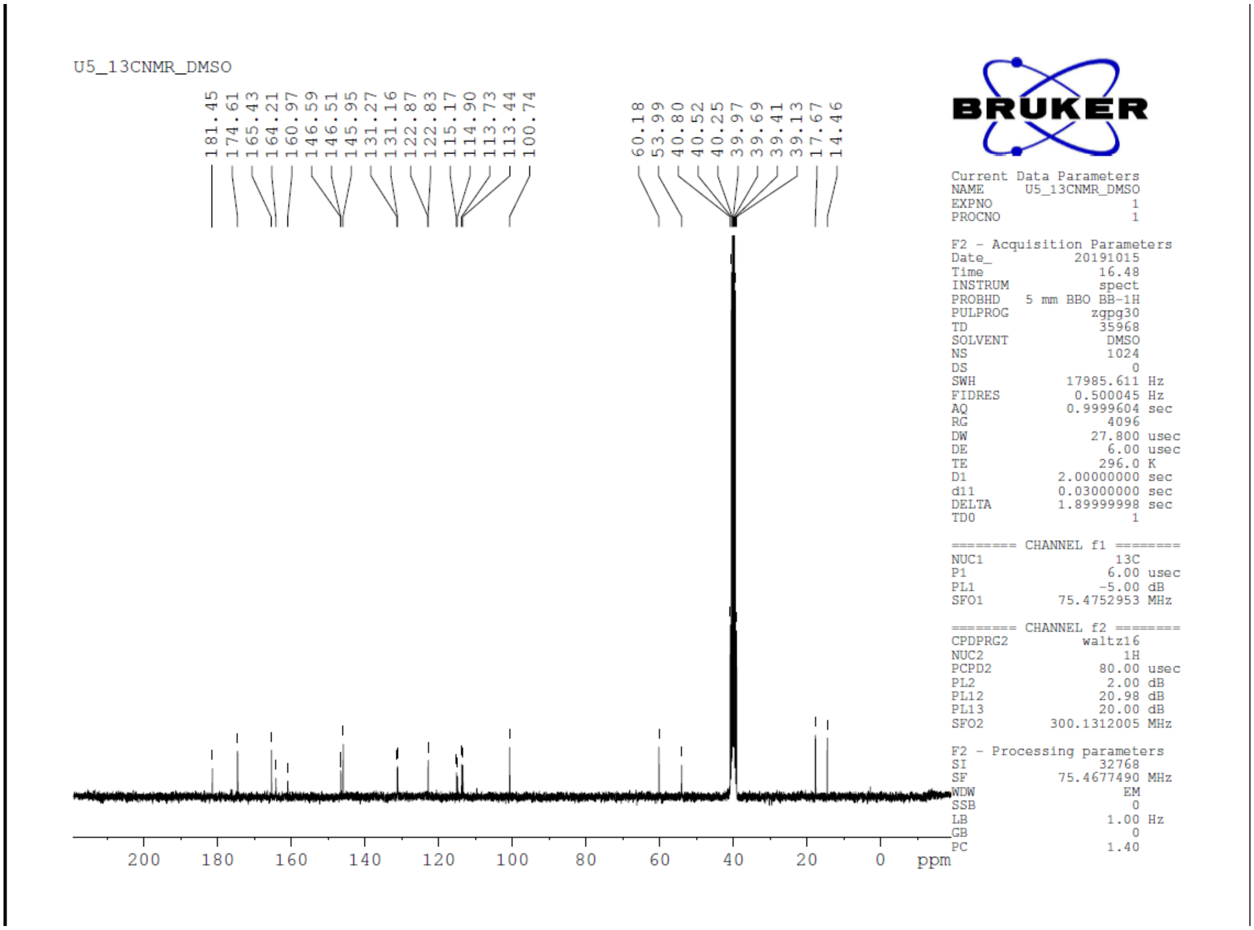

Figure. S12 ^{13}C-NMR spectra of 5e.

6. NMR spectra of ethyl 4-(4-fluorophenyl)-6-methyl-2thioxo-1,2,3,4-tetrahydropyrimidine-5-carboxylate (5f, $C_{14}H_{15}FN_2O_2S$):

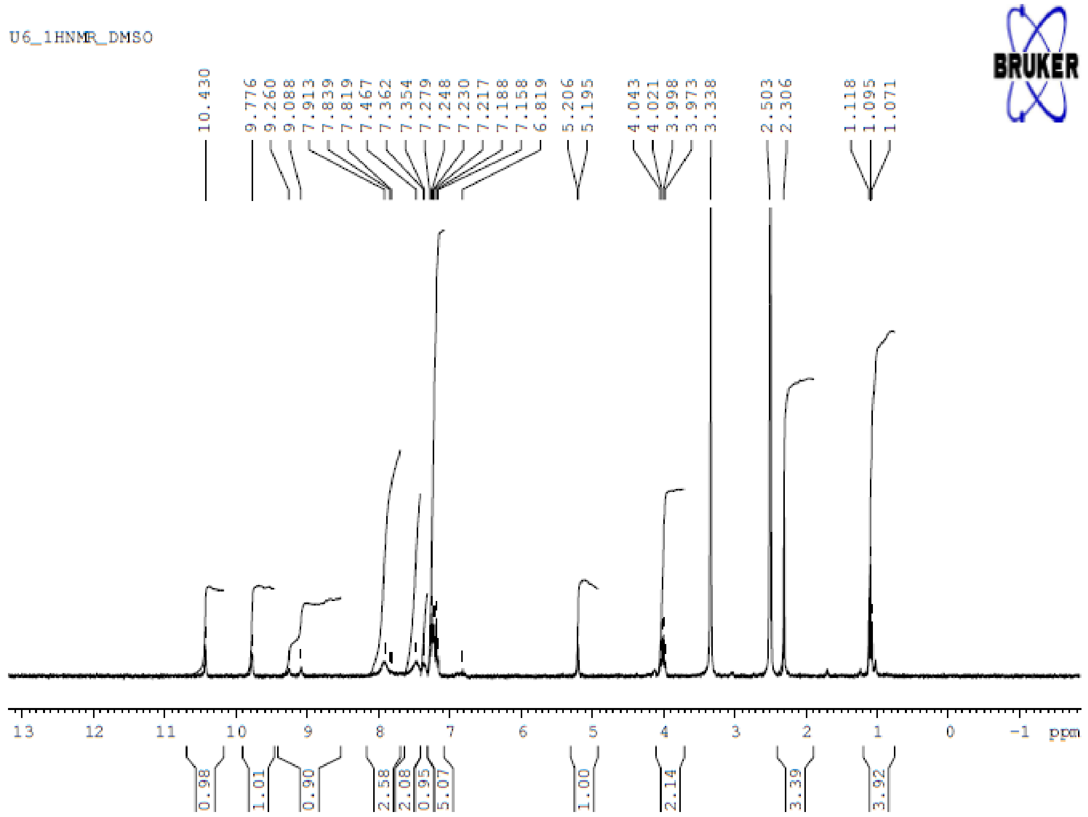

Figure. S13. ^{1}H-NMR spectra of 5f.

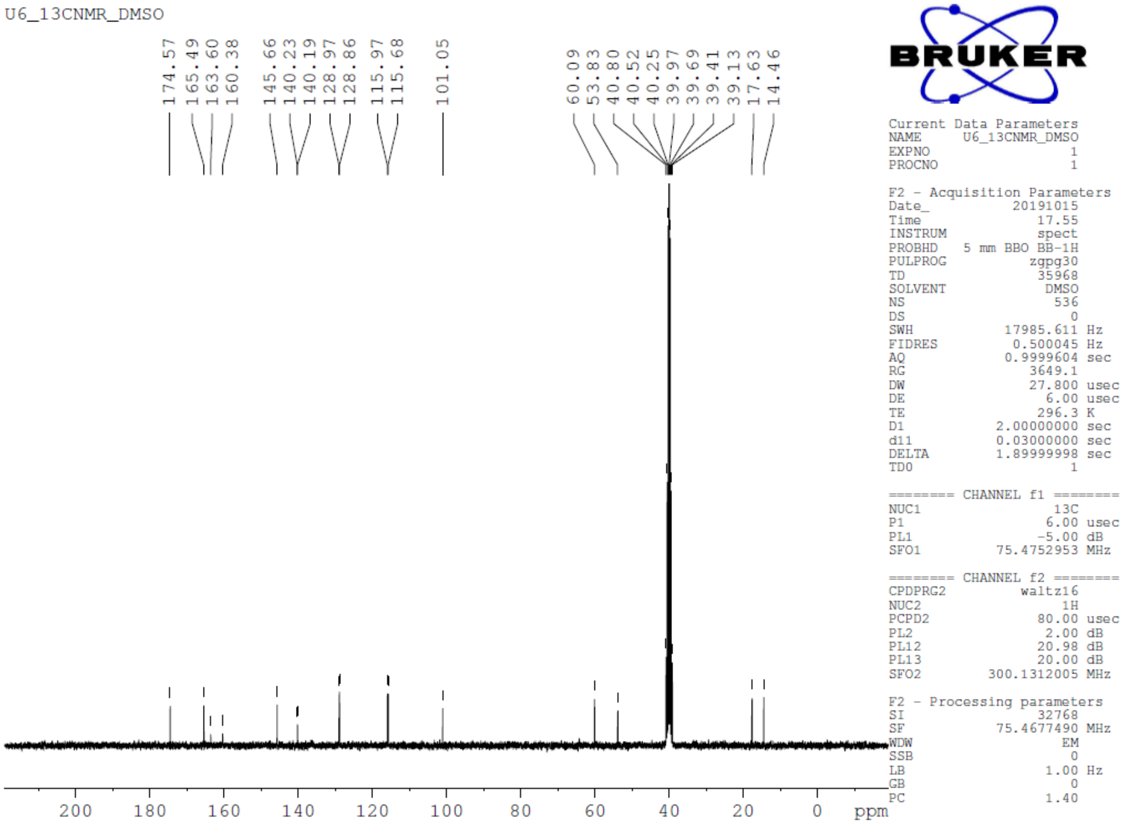

Figure. S14. ^{13}C-NMR spectra of 5f.

7. NMR spectra of ethyl 4-(3-chlorophenyl)-6-methyl-2oxo-1,2,3,4-tetrahydropyrimidine-5-carboxylate (5g, $C_{14}H_{15}ClN_2O_3$):

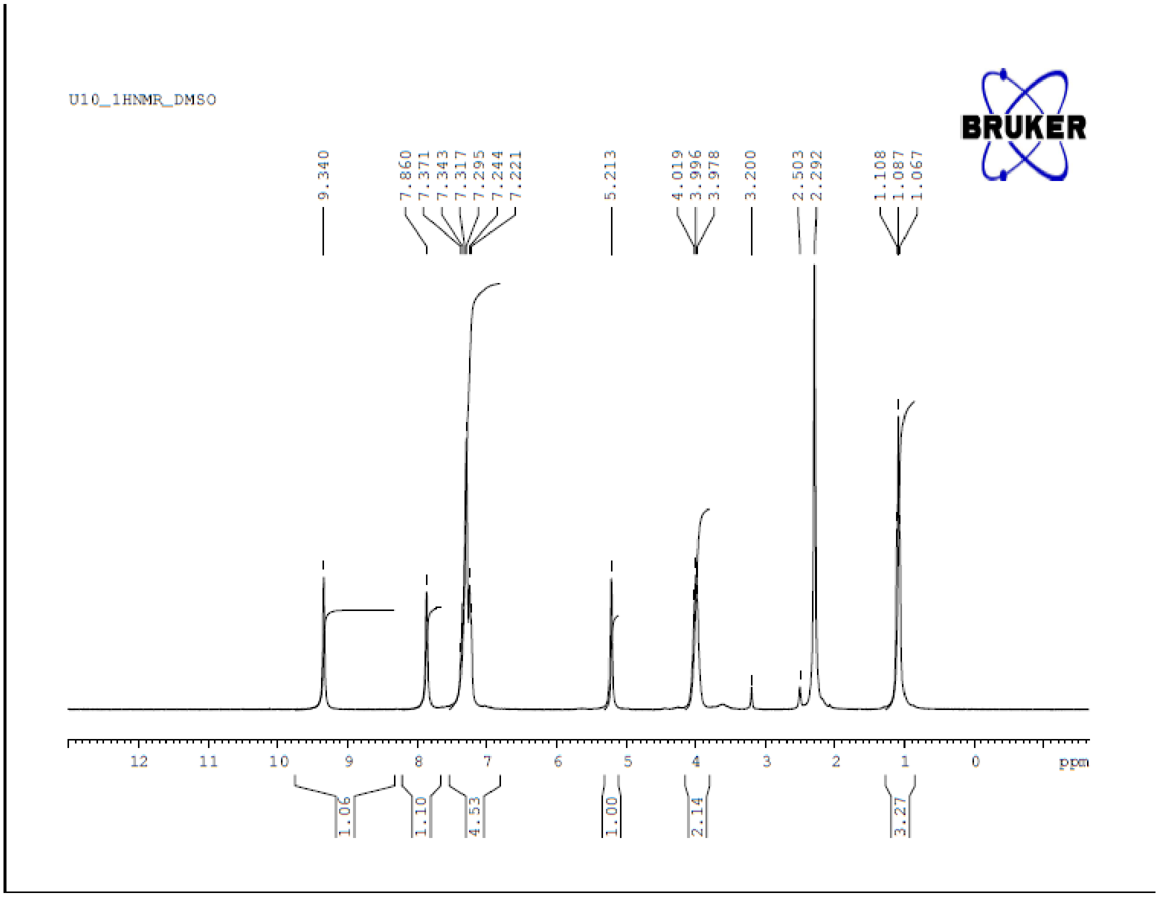

Figure. S15. ^{1}H-NMR spectra of 5g.

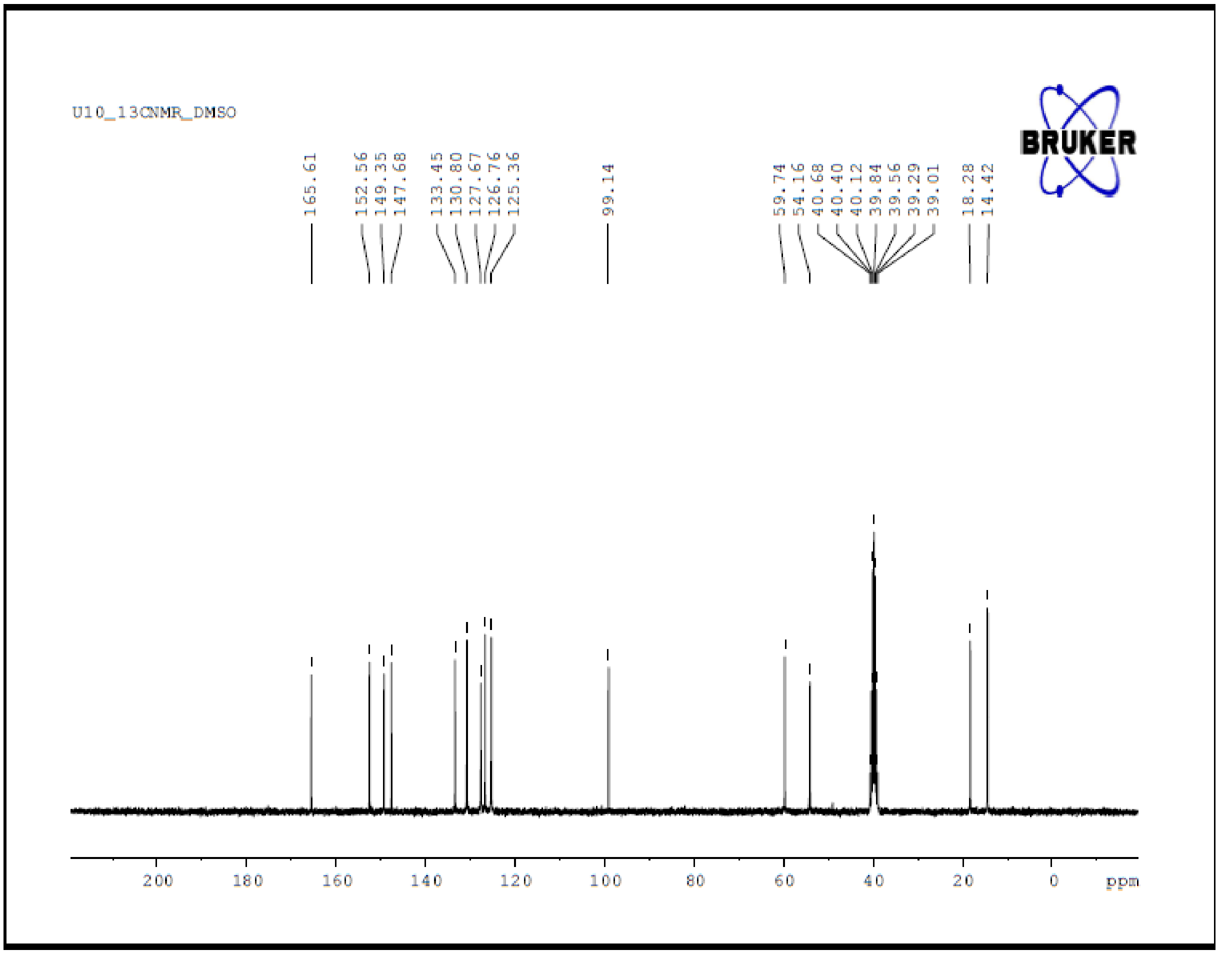

Figure. S16. ^{13}C-NMR spectra of 5g.

8. NMR spectra of ethyl 4-(3-chlorophenyl)-6-methyl-2thioxo-1,2,3,4-tetrahydropyrimidine-5-carboxylate (5h, $C_{14}H_{15}ClN_2O_2S$)

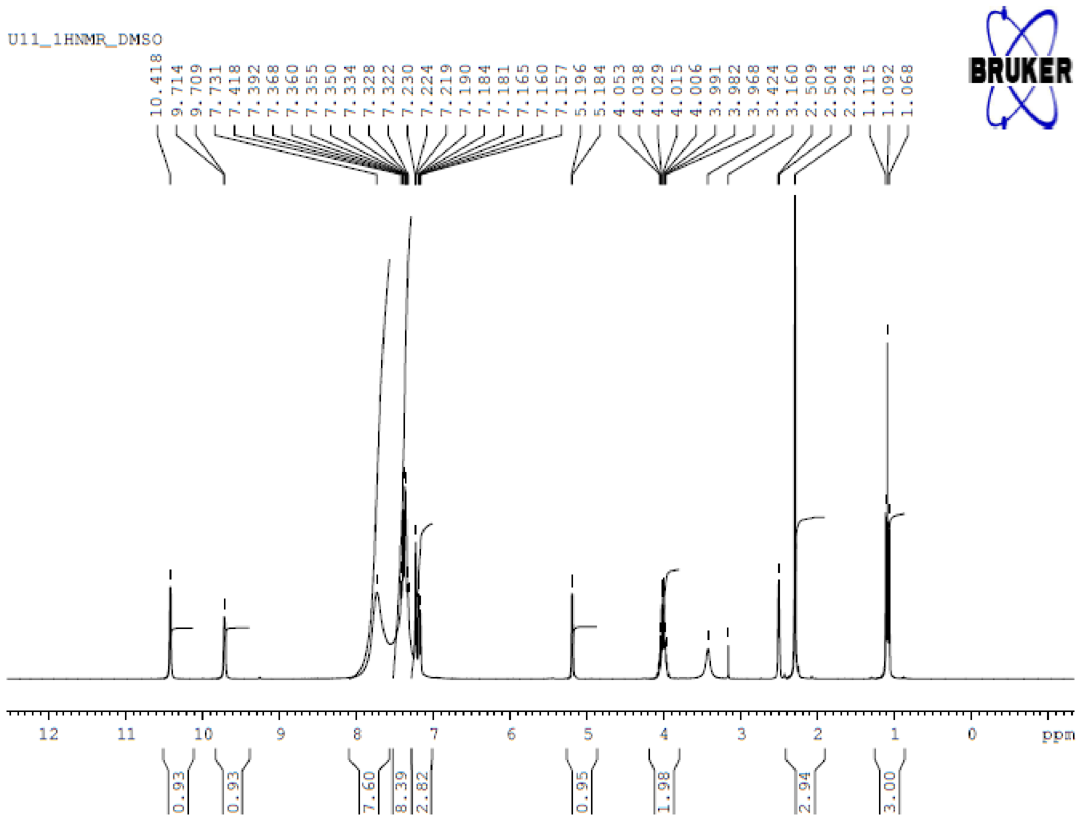

Figure. S17. ^{1}H-NMR spectra of 5h.

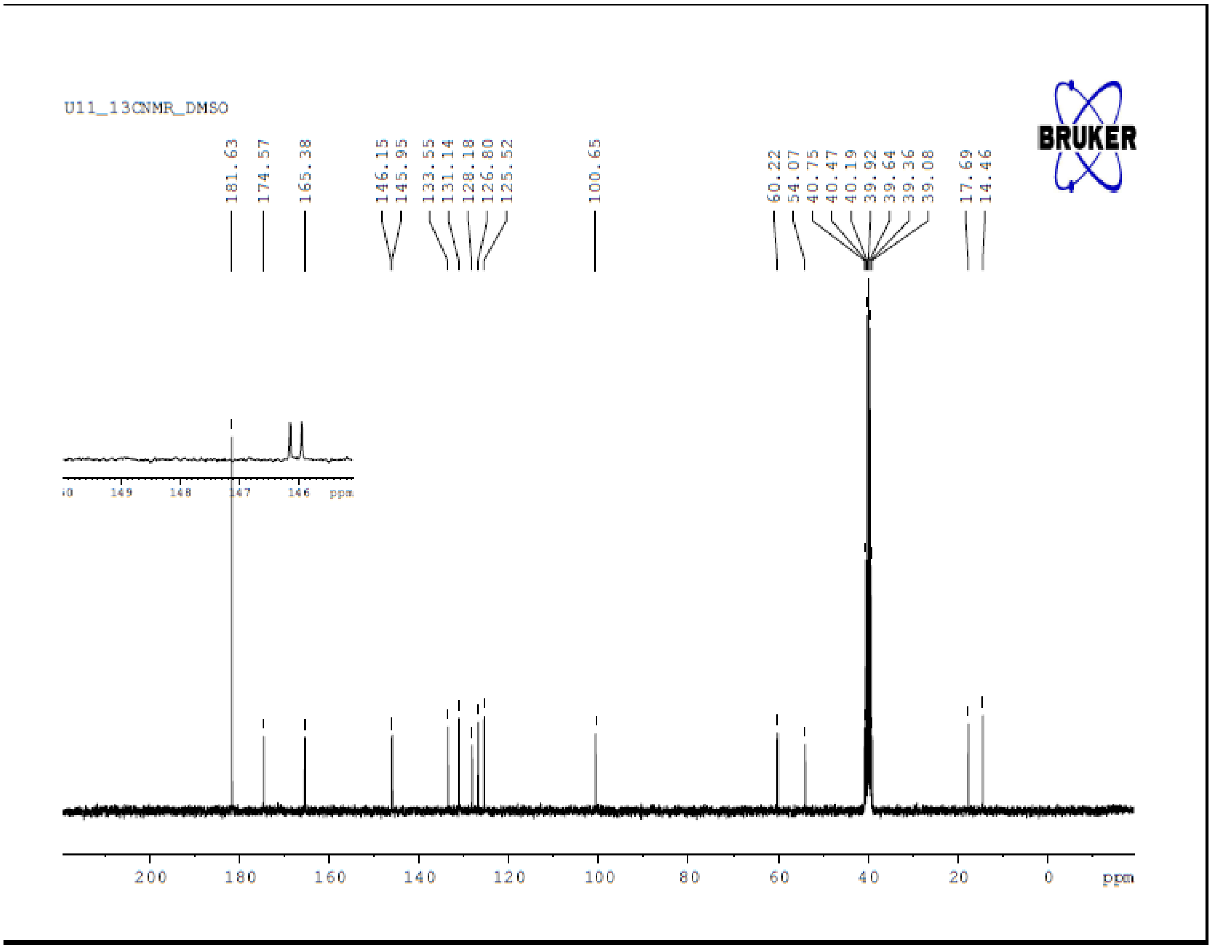

Figure. S18. ^{13}C-NMR spectra of 5h.
